# Gain of Function Notch Phenotypes Associated with Ectopic Expression of the Su(H) C-Terminal Domain Illustrate Separability of Notch and Hairless-Mediated Activities

**DOI:** 10.1371/journal.pone.0081578

**Published:** 2013-11-25

**Authors:** Dieter Maier, Heiko Praxenthaler, Adriana Schulz, Anette Preiss

**Affiliations:** Universität Hohenheim, Institut für Genetik (240), Stuttgart, Germany; Institut Pasteur, France

## Abstract

The Notch signaling pathway is instrumental for cell fate decisions. Signals from the Notch receptor are transduced by CSL-type DNA-binding proteins. In *Drosophila*, this protein is named Suppressor of Hairless [Su(H)]. Together with the intracellular domain of the activated Notch receptor ICN, Su(H) assembles a transcriptional activator complex on Notch target genes. Hairless acts as the major antagonist of the Notch signaling pathway in *Drosophila* by means of the formation of a repressor complex together with Su(H) and several co-repressors. Su(H) is characterized by three domains, the N-terminal domain NTD, the beta-trefoil domain BTD and the C-terminal domain CTD. NTD and BTD bind to the DNA, whereas BTD and CTD bind to ICN. Hairless binds to the CTD, however, to sites different from ICN. In this work, we have addressed the question of competition and availability of Su(H) for ICN and Hairless binding in vivo. To this end, we overexpressed the CTD during fly development. We observed a strong activation of Notch signaling processes in various tissues, which may be explained by an interference of CTD with Hairless corepressor activity. Accordingly, a combined overexpression of CTD together with Hairless ameliorated the effects, unlike Su(H) which strongly enhances repression when overexpressed concomitantly with Hairless. Interestingly, in the combined overexpression CTD accumulated in the nucleus together with Hairless, whereas it is predominantly cytoplasmic on its own.

## Introduction

In multicellular organisms the Notch signaling pathway plays a pivotal role during development and homeostasis, allowing cell to cell communication. As a consequence of Notch signaling activity, cells will adopt a different cell fate (reviewed in 1). Signaling is initiated upon the binding of two transmembrane proteins presented by neighboring cells: in the receiving cell the receptor Notch and in the sending cell the ligand DSL (Delta, Serrate, Lag2). Activation of the canonical Notch signaling pathway is well understood: DSL-binding of Notch results in its intracellular cleavage and release of the intracellular domain (ICN). ICN migrates to the nucleus, binding to the transcription factor CSL (reviewed in 1-3). The CSL acronym is derived from the human homolog CBF1, from *D. melanogaster* Suppressor of Hairless [Su(H)], and from *C. elegans* Lag1. CSL molecules are highly conserved: they consist of three domains, the N-terminal (NTD), the beta-trefoil (BTD) and the C-terminal (CTD) domain. Together, the NTD and the BTD bind sequence specifically to the DNA of Notch target gene promoters [4,5]. By binding to the BTD and the CTD, ICN assembles an activator complex together with other co-activators [6,7] (reviewed in 8). 

In vertebrates and in *Drosophila* this process is antagonized by proteins which transform CSL into a transcriptional repressor of the Notch target genes (reviewed in 2,9). In vertebrates in the absence of Notch signaling, CBF1 recruits several different co-repressors that all bind to the BTD thereby competing with ICN (reviewed in 2,9). In *Drosophila* downregulation of Notch signaling activity is likewise a consequence of direct repression of the Notch target genes: in this case a repressor complex consistent of Su(H) and the major Notch antagonist Hairless plus several co-repressors is assembled [10,11] (reviewed in 3,12). Hairless binds to the CTD of Su(H), however, to sites different from ICN and in fact, has little capacity to compete with ICN [13]. Su(H) may be therefore regarded as a molecular switch, and activation like repression is taking place on the DNA with Su(H) at the heart of either process (reviewed in 9,12,14). In this model, ICN and Hairless compete for Su(H) while sitting on the DNA. A strong Notch signal may release enough ICN to replace Hairless from Su(H), and target gene activation starts ([11,15]; reviewed in 9,12,14). 

There is mounting evidence, however, that this picture is incomplete, and probably not all of Su(H) regulation takes place at the level of DNA. The most direct evidence for a more complex Su(H) regulation comes from work studying its distribution with regard to signal activation. Here it was shown that Notch target gene promoters are not permanently occupied by Su(H) in the absence of Notch signaling suggesting that the repressor complexes are as transient as the activator complexes ([16]; reviewed in 3). Moreover, CSL itself has no typical nuclear localization signal and hence its nuclear import is dependent on other factors [17-21], suggesting an additional layer of regulatory input. Mammalian CBF1 is predominantly nuclear, whereas *Drosophila* Su(H) is found in the cytoplasm and the nucleus [17-20,22,23]. Despite of this difference, in either system both ICN and co-repressors may serve the nuclear transport of CSL. For example, in *Drosophila* it was shown that Su(H) is only found in the nucleus in the presence of ICN [17,18]. In human cell culture, transformation with ICN results in a stable high molecular weight activator complex containing amongst others CBF1 and ICN within the nucleus [24]. In addition, the SMRT co-repressor regulates nuclear entry of CBF-1 [20]. Similarly, Su(H) colocalizes with Hairless in the cytosol and in the nucleus: In the absence of Hairless, Su(H) appears less abundant, whereas in response to ectopic Hairless expression Su(H) is more enriched in the nucleus [19]. These findings strongly suggest that the exchange of activator to suppressor complex and vice versa is not restricted to DNA-bound CSL, in accordance with the transient occupancy of Notch promoters by Su(H) [16]. Hence in addition to the active processes at the level of DNA, there might be as well passive processes that result in an activation or repression of Notch signaling activity. For example, repression may occur by interference with Su(H) availability as a result of the binding of Hairless or Notch that happens distant from the DNA. 

In this work we ectopically expressed a Su(H) construct consisting only of the CTD of Su(H). This region was shown to bind to Hairless as well as to the Ankyrin repeats of ICN, however at different sites [13]. As a consequence of the overexpression, a gain of Notch activity was obtained comparable to that of an overexpression of full length Su(H). Since CTD cannot bind to the DNA, any activation must rely on a ‘passive mode’. Accordingly, a combined overexpression of CTD together with Hairless ameliorated the effects, unlike Su(H) which strongly represses Notch signaling output when overexpressed together with Hairless [11,13,15]. We propose that ectopic CTD traps endogenous Hairless, thereby limiting repressor complex formation on target gene promoters. Interestingly, in the combined overexpression CTD accumulates in the nucleus together with Hairless, whereas it is predominantly cytoplasmic on its own. In sum this work supports the idea that CTD and Hairless can form sterile complexes in the cytoplasm as well as in the nucleus, curbing Hairless activity and hence resulting in an increased Notch signaling output. 

## Materials and Methods

### Generation of myc-CTD and RICN constructs

The CTD, representing codons 417-528, was PCR amplified from a Su(H) cDNA [25] and cloned via *Eco* RI / *Xho* I sites provided by the primers into the pBT-vector (Stratagene, La Jolla CA, USA) [13]. After opening the pBT-CTD subclone with a *Bam* HI / *Eco* RI digest, a myc-tag was added in frame 5-prime to the CTD with annealed primers that provided the respective sticky ends, and included a *Bgl* II site for verifying the subclone. The myc-CTD construct was shuttled as *Bam* HI / *Xho* I fragment into *Bgl* ll / *Xho* I digested pUAST-attB vector; transgenic flies were established with the PhiC31 (96E) strain [26]. The integration was confirmed by PCR. For cell culture assays myc-CTD was excised with *Eco* RI / *Kpn* I from the pUAST-attB vector and cloned into likewise opened pRmHa-3 vector [27] to generate pMT-myc-CTD. 

RICN (intracellular Notch containing the RAM domain) was PCR amplified from a Notch cDNA clone [28] with primers starting from base 6057 and ending at base 9019 (numbering is according to Flybase, http://flybase.org). The upper primer included a *BgI* II and the lower primer a *Xba* I site allowing the amplified RICN DNA to be cloned into *Bam* HI / *Xba* I restricted shuttle vector and to be transferred subsequently via *Acc* 65I / *Xba* I into likewise digested pUAST-attB vector for transformation. The translation starts at an internal methionine at position 1762 and ends at 2703 with the normal Notch STOP codon. All constructs were sequence verified (StarSeq, Mainz, Germany). Primer sequences and cloning details are available upon request.

### Cell culture assays

The assays were performed as described earlier [13,29,30] using Schneider S2 cells, obtained from the Drosophila Genomics Resource Centre DGRC (Indiana University, Bloomington USA). The cells were transfected with 1 µg of the Notch response element (NRE) [29], 1 µg of pMT-ICN [31] plus 0.5 µg of pMT-Su(H) and / or 0.5 µg of pMT-HFL, and / or 0.5 or 0.1 µg of pMT-myc-CTD as indicated, and 0.2 µg of pRL-TK as internal standard (TK-Renilla; Promega, Madison WI, USA), normalized to 5 µg with pMT-A (Invitrogen, Carlsbad CA, USA). To activate expression of the pMT constructs 0.5 mM CuSO_4_ was added 6h after transfection. The luciferase activity was measured 18h later in duplicate (Lumat LB 9507; EG&G, Salem MA, USA) using the dual-luciferase reporter gene assay system (Promega Corp., Mannheim, Germany). Three independent experiments were performed and the data sampled.

### Generation and analysis of transgenic flies

Transgenic lines were generated using the PhiC31 integrase system [26]. Myc-CTD was inserted into the Φ-96E site to allow a direct comparison with Su(H) that is integrated at the same position [13]. Myc-CTD (96E) was recombined with Hairless HFL (68E), and the recombinant was compared with the HFL (68E) Su(H) (96E) recombinant as described before [13]. In addition Su(H) and Su(H)^E446K^ were inserted into the Φ-22A site, and the RICN construct into the Φ-58A site. Recombination yielded Su(H) (22A) RICN (58A). Transgenic lines and recombinants were tested by PCR and by antibody detection of the respective proteins upon overexpression. The pUAST-dsH line is described in [32]. UAS-GFP was used as control.

Tissue specific expression of transgenes was achieved with the Gal4-UAS system [33]. Crosses with omb-Gal4 and Bx-Gal4 (also named MS1096-Gal4) were reared at 18°C, crosses with prd-Gal4 and gmr-Gal4 at 25°C; driver lines are described in Flybase (http://flybase.org). The vg^BE^-lacZ line [34] was used to study expression of the Notch target gene *vestigial* (*vg*). Flies and wings were photographed with an ES120 camera (Optronics, Goleta CA, USA) using Pixera Viewfinder software, version 2.0, and with a table-top scanning electron microscope (Neoscope JCM-5000; Nikon, Tokyo, Japan). 

### Immunohistochemistry

For the analysis of respective protein and reporter gene expression, the following primary antibodies were used as described before [13,30]: guinea pig Hairless anti-A and rabbit Hairless anti-NTH [19,35]; anti-Su(H) made in rat (Pineda, Berlin, Germany) using the Su(H) (288-594) GST-protein [18]; rabbit anti-myc A4-1 (Santa Cruz Biotechnology, Santa Cruz, CA); mouse anti-π-myc (gift from S. Artavanis-Tsakonas); mouse anti-beta-galactosidase (developed by J.R. Sanes) and mouse anti-Notch intracellular domain (developed by S. Artavanis-Tsakonas) (both from DSHB Developmental Studies Hybridoma Bank, developed under the auspices of the NICHD and maintained by the University of Iowa, Dept. of Biology Iowa City IA, USA). Secondary antibodies coupled with DTAF or Cy3 were purchased from Jackson Immuno-Research Laboratories (Dianova, Hamburg, Germany). Imaginal discs and embryos were mounted in Vectashield (Vector Labs, Biozol, Eching, Germany) and analyzed on a Zeiss Axiophot linked to a Bio-Rad MRC1024 confocal microscope (Zeiss, Jena, Germany). 

For immunoprecipitations, batches of 100 fly heads with the genotype gmr-Gal4 / +; UAS-HFL UAS-myc-CTD / + were homogenized in RIPA buffer (150 mM NaCl, 50 mM Tris-HCl pH 7.5, 1% Triton X100, 0.1% SDS, 1 complete Mini protease inhibitor cocktail [Roche, Mannheim, Germany]) and incubated over night in 10 mM Tris-HCl pH 7.5 plus protease inhibitors with rabbit anti-myc antiserum (1:40). 40 µl of protein A beads (Roche, Mannheim, Germany) were added, incubated for 4 hours and washed several times. The mock control was without antibodies. All steps were performed on ice. Beads were collected by centrifugation and boiled in loading dye, 30% of which was separated on SDS-PAGE and proteins detected on a Western blot together with 15% of the input with guinea pig anti-Hairless A and rabbit anti-myc antibodies, respectively. 

## Results and Discussion

### Overexpression of CTD causes Notch gain of function phenotypes

**Figure 1:**
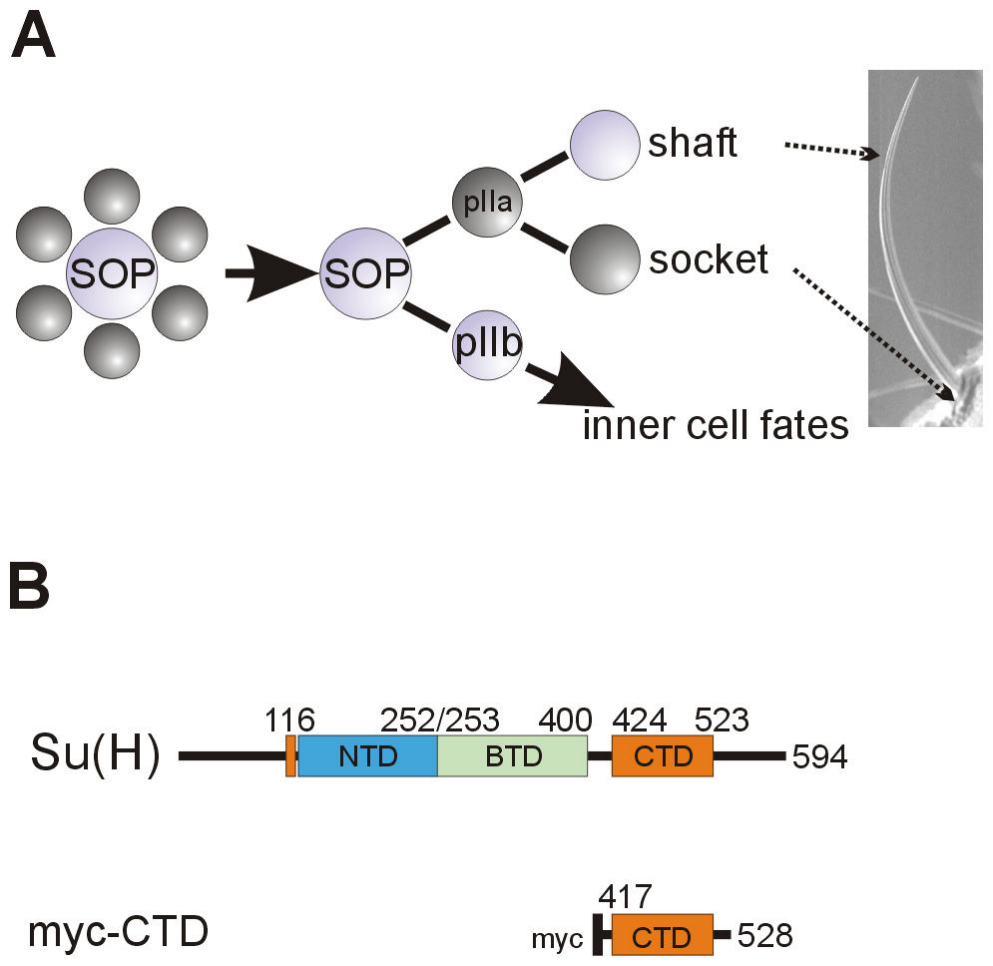
Scheme of bristle development and the myc-CTD construct. A) During bristle development, a sensory organ precursor cell (SOP) is singled out by lateral inhibition from a cluster of equipotential, proneural cells. By activating the Notch pathway, the SOP forces the surrounding cells into a secondary fate (labeled dark grey). The SOP divides asymmetrically, unequally activating the Notch pathway in the daughter cells: the pIIa cell receives a Notch signal and gives rise to the outer cell lineage, whereas inner cell fate is derived from pIIb. The pIIa daughter that receives a Notch signal will form the socket, the other daughter cell will form the bristle shaft (according to [36,38]). B) The Su(H) protein consists of three highly conserved domains, the N-terminal domain (NTD, blue), the β-trefoil domain (BTD, green) and the C-terminal domain (CTD, orange). The N-terminal helix (orange) is in the proximate neighborhood of the CTD in the three-dimensional structure [7,8]. The numbers represent the amino acids of the protein. In the CTD construct, codons 417 to 528 where fused to a myc coding sequence providing the start methionine and the myc-tag for antibody staining (black).

**Figure 2:**
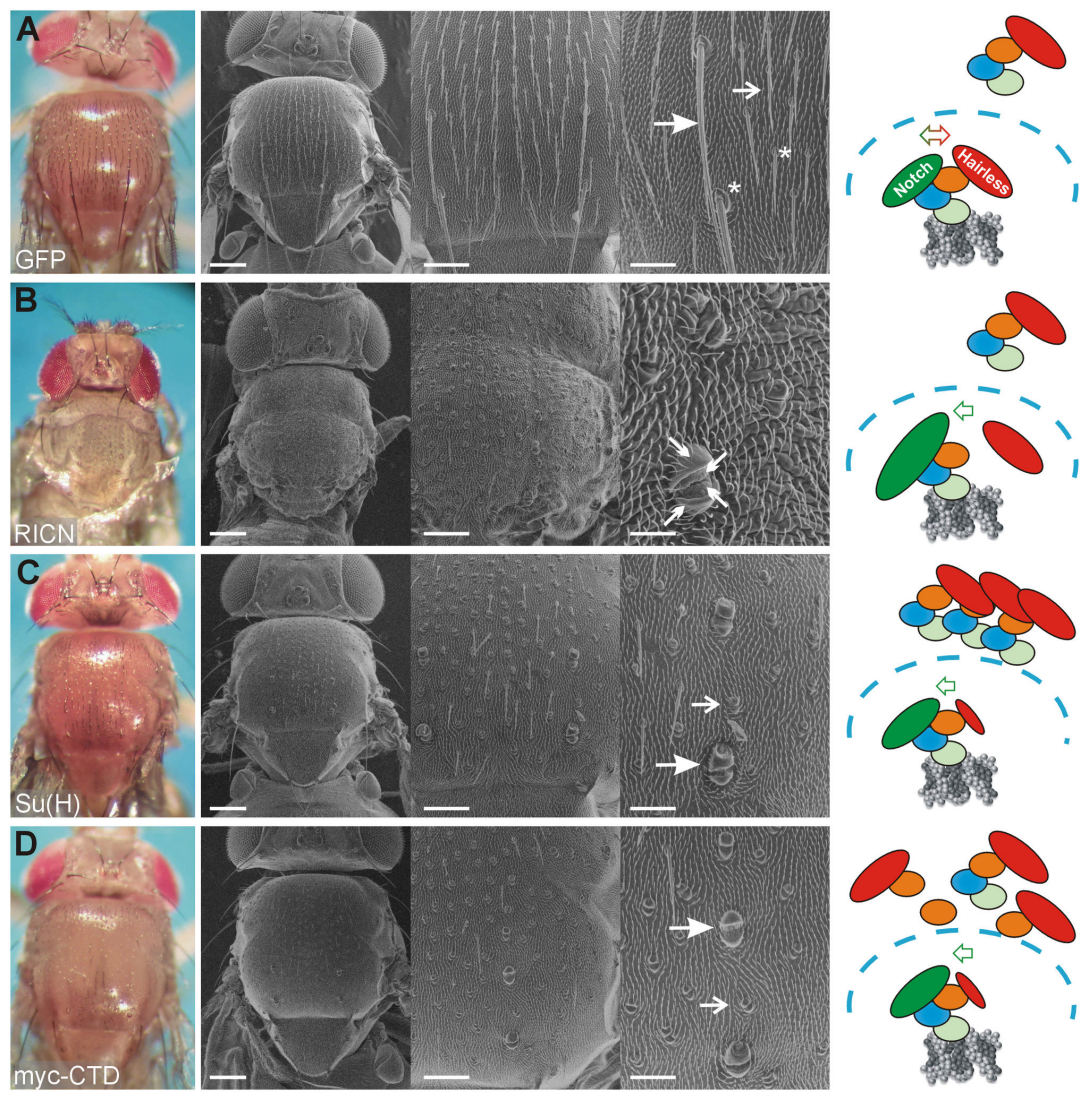
Overexpression of CTD causes Notch gain of function phenotypes. A) Overexpression of GFP results in wild type looking flies and was used as control. The enlargement highlights the microchaetae (open arrow), macrochaetae (closed arrow), bristle shafts and sockets (asterisk). The cartoon depicts the relevant molecules: Su(H) in blue, green and orange reflecting the NTD, BTD and CTD, respectively is cytoplasmic and nuclear (dashed line), where it binds to the DNA (grey). Hairless (red) is bound to Su(H) in either compartment. ICN (green) is bound to nuclear Su(H) on the DNA. Hairless and ICN are balanced in the wild type: activation and repression take place on the DNA (red/green double-headed arrow). B) Overexpression of the activated Notch intracellular domain (UAS-RICN) causes a transformation of inner into outer fate and shaft into socket fate, giving rise to double and quadruple sockets (see four arrows in enlargement). Nearly all bristles are affected. As Notch overexpression affects multiple tissues, the flies die as pharate adults. In the cartoon, ectopic ICN is shown as enlarged circle that directly outcompetes endogenous Hairless, thereby shifting signaling into the activation mode. C) Overexpression of Su(H) is shown for comparison. It causes a transformation of shaft into socket cells affecting the majority of micro- and macrochaetae (open and closed arrows). The cartoon depicts the ectopic Su(H) molecules in both, the nuclear and the cytoplasmic compartments, where they may form additional activator complexes or curb Hairless activity, respectively, shifting the balance in the active mode. D) The myc-CTD transgene likewise enforces a shaft to socket cell transformation, affecting macro- and microchaetae alike (open and closed arrows). As highlighted in the cartoon, CTD cannot bind to the DNA, hence its impact on Notch target gene activity must be indirect. CTD may trap Hairless in the cytoplasm, reducing its availability in the nucleus and shifting the balance into an active mode. PHOTOs on the left were taken with the ES120 camera (colored).

Notch signaling is well known to control the process of lateral inhibition which can be exquisitely studied during sensory organ development of the fly (for review see 36,37. Sensory organs derive from precursor cells (SOPs) and their number is restricted by Notch signaling. Each SOP develops after a total of four asymmetric divisions into a bristle consisting of two outer cells, the shaft and the socket, and three inner cells including the neuron [38] (sketched in Figure 1A). Eventually, the adult fly is decorated with evenly spaced microchaetae and precisely positioned macrochaetae (reviewed in 36,37) (see Figure 2A). It was shown in the past that the Notch signaling pathway regulates this developmental process at every single step. Changes in the activity of Notch pathway members, either by mutation or by ectopic expression, influence the cell fate decisions. As a consequence the cells adopt a wrong cell fate (reviewed in 36). Therefore the bristle development is a commonly used process to study the activity of the Notch signaling pathway (e.g. 18,25,28,38-55). For example, hyperactivity of Notch signaling was reported to result in a transformation of inner to outer cell fates and shaft into socket cells. We have generated a new Notch ICN construct (UAS-RICN, consistent of ICN plus the complete RAM domain) that was overexpressed in the developing thorax and head using the Bx-Gal4 driver line. As expected from the earlier reports [28,40,42,46,54], this overexpression resulted in double and quadruple sockets instead of a normally formed bristle (Figure 2B). Compared to the wild type, where ICN mediated activator complexes and Hairless mediated repressor complexes are balanced (Figure 2A), the overexpression of ICN entails a shift towards activator complexes, accompanied by cell fate changes (Figure 2B). A similar, albeit quantitatively weaker phenotype is obtained in response to the overexpression of Su(H) under the same conditions. In accordance with earlier data [50,53,54], we observed a nearly complete transformation of shaft into socket cells in response to the overexpression of our UAS-Su(H) construct with the Bx-Gal4 driver line (Figure 2C), demonstrating a gain of Notch signaling activity. Apparently, raising the amount of Su(H) shifts the balance in the activation mode. The simplest explanation is that Su(H) outcompetes the repressor Hairless, causing increased Notch activity in a passive manner (Figure 2C). However, Su(H) has a dual function: it can either bind ICN plus co-activators or it can bind Hairless plus co-repressors (reviewed in 3,9,12). Hence, apart from building up additional activator complexes, ectopic Su(H) is expected to trap endogenous Hairless protein, thereby shifting the balance from repressor to activator complexes (Figure 2C).

In grey are scanning electron micrographs with increasing magnification from left to right; scale bars in A, C, D) 200 µm, 100 µm and 50 µm and in B) 200 µm, 100 µm and 20 µm, respectively. Genotypes are: (A) Bx-Gal4 / +; UAS-GFP / +, (B) Bx-Gal4 / +; UAS-RICN / +, (C) Bx-Gal4 / +; UAS-Su(H) / + and (D) Bx-Gal4 / +; UAS-myc-CTD / +. 

To study a possible passive regulation of the Notch signaling pathway, a construct was generated that covers the C-terminal domain of Su(H) (CTD) (Figure 1B). CTD contains the binding domain for the Notch Ankyrin repeats as well as for Hairless but has no DNA binding capacity [4,5,13,30]. It was fused upstream with coding sequences of a myc-tag (myc-CTD; Figure 1B), cloned under the control of UAS-sequences and inserted in the fly genome at the same site as the wild type Su(H) construct using the PhiC31 system (see Material and Methods). Interestingly, the overexpression of myc-CTD caused a qualitatively similar phenotype than the overexpression of wild type Su(H) protein, i.e. a gain of Notch signaling activity (compare Figure 2C and 2D). A transformation of shaft into socket cells resulting in a double socket phenotype was seen affecting nearly all micro- and macrochaetae alike (Figure 2D). Since both constructs are at the identical genomic location (96E), position effects can be excluded, and the two transgenes can be directly compared [13,26]. Our working hypothesis for the observed overstimulation of Notch signaling is a weakening of Hairless repressor activity by the overexpression of CTD. Lacking a DNA binding domain, the CTD cannot directly repress Notch target gene expression when bound to Hairless. Even if a complete repressor complex is formed by CTD, Hairless plus co-repressors, it cannot assemble on the DNA of Notch target gene promoters. Therefore we suggest a passive inhibitory function for CTD: by trapping Hairless in the cytoplasm and/or in the nucleus, availability of Hairless and hence formation of repressor complexes is reduced (Figure 2D), thereby shifting the system into an active mode.

To study a different developmental context, we compared the effects of myc-CTD overexpression with that of either Su(H) or Hairless in the developing eye. To this end we used the GMR-Gal4 line which drives expression in the differentiating eye field (see supporting Figure S1). An enforced Notch signaling activity is known to result in a marked overproliferation of the eye (see e.g. 56-58), whereas overexpression of Hairless causes the contrary phenotype due to cell death (supporting Figure S1) (see also 57,59-61). Similar to its effect during bristle development, ectopic expression of myc-CTD also caused enlarged eyes, but less pronounced than with ectopic expression of Su(H) (supporting Figure S1). 

The effects of CTD overexpression were also compared with that of Su(H)^E446K^. In this mutant, glutamic acid at position 446 is exchanged by a lysine, strongly interfering with ICN binding [13]. However, neither binding to Hairless nor to the DNA is affected [13]. Hence Su(H)^E446K^ is formally similar to CTD as it is expected to be able to outcompete Hairless and hence to cause likewise Notch gain of function effects. But in contrast to CTD, Su(H)^E446K^ can assemble repression complexes together with Hairless on the DNA, whereas activator complex formation is presumably impaired based on the lack of ICN binding [13]. In fact, in response to the overexpression of Su(H)^E446K^ a partial shaft to socket transformation was observed typical of a subtle Notch gain of function (supporting Figure S2). 

### Overexpression of CTD ameliorates Hairless gain of function phenotypes in vivo

**Figure 3:**
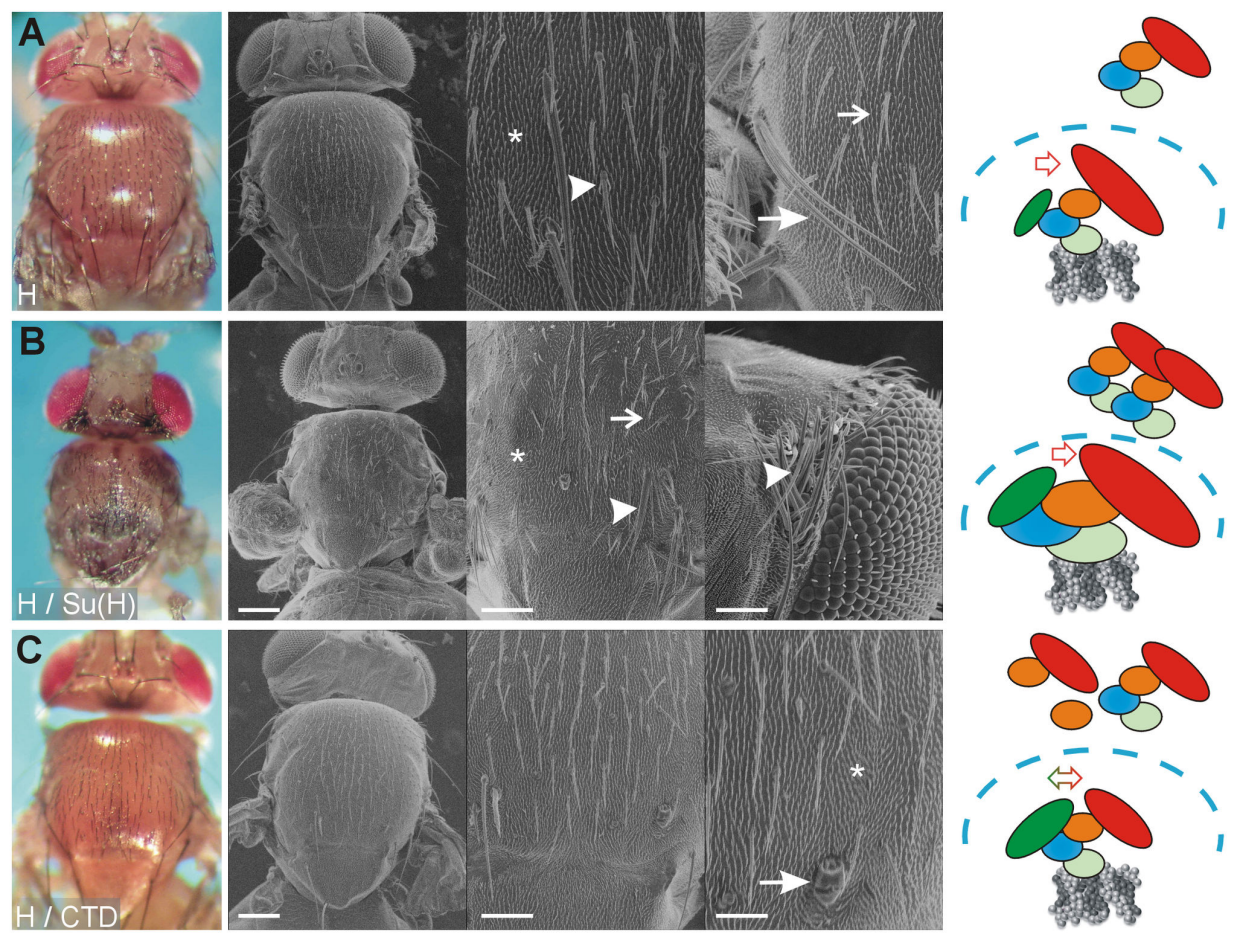
CTD cannot form a super-repressor. A) The consequences of an overexpression of Hairless (H), shown as a reference, are double shafts mostly affecting microchaetae (open arrow) and more rarely macrochaetae (closed arrow). Note also partial transformation of microchaetae (arrowhead). Bristle loss is also observed (asterisk) and can be explained by a transformation of outer into inner cell fates (pIIa to pIIb, see Fig. 1A). The cartoon depicts ectopic Hairless enlarged. By replacing activator with repressor complexes Hairless enforces a repressive mode. B) Combined overexpression of Su(H) and Hairless (H) is shown as a reference. It causes a super-repressor phenotype which reflects a strong loss of Notch activity [11,15]. In addition to double shafts (open arrow) and bristle loss (asterisk), bushes of bristles are observed (arrowhead) that reflect a collapse of the lateral inhibition process. Note that the flies die before eclosion as pharate adults. In the cartoon, the super-repressors are shown enlarged, as is the shift in the repression mode. C) Combined overexpression of myc-CTD with Hairless (H) subtly disturbs bristle development: a double socket points to a Notch gain of function (arrow), whereas bristle loss to a Hairless gain of function (asterisk), as does the slightly higher bristle density. The cartoon depicts the model that ectopic CTD is able to quench the effects of ectopic Hairless, as expected if the two bound already in the cytosol. PHOTOs on the left were taken with the ES120 camera (colored).

Notch signaling is obstructed by the overexpression of Hairless [40,46,54]. As expected from earlier reports [39,40,48-50], a transformation of sockets into shafts as well as of outer into inner cell fates was observed, resulting in double shafts and bald cuticle (Figure 3A). Apart from assembling repressor complexes, ectopic Hairless may as well compete with ICN for endogenous Su(H), thereby shifting the system into the repression mode (Figure 3A). An extreme downregulation of Notch activity can be enforced by a combined overexpression of Hairless and Su(H), which has been observed before in systemic as well as tissue specific induction [11,13,15,32]. An example for a combined expression during head and thorax development is shown in Figure 3B and in supporting Figure S1. Overexpression was performed under the same conditions in parallel with the single overexpression experiments to allow for a direct comparison (Figures 2B, C, and 3A, B). As noted before [11,13], all Notch dependent steps of bristle formation were strongly affected, asymmetric cell divisions as well as lateral inhibition. Note bald patches, double bristles and bristle clusters notably on the head that reflect a collapse of lateral inhibition (Figure 3B). Likewise strong effects were seen during eye development: the eye was almost completely absent and was without any visible external structures that typify the *Drosophila* eye (supporting Figure S1). This result has been interpreted before to be a consequence of the formation of a powerful Su(H)-Hairless repressor complex that massively inhibits Notch target gene expression [11,13,15,32].

In grey are scanning electron micrographs with increasing magnification from left to right; scale bars in A) 200 µm, 50 µm and 50 µm; and B-C) 200 µm, 100 µm and 50 µm, respectively. Genotypes are: (A) Bx-Gal4 / +; UAS-HFL / +, (B) Bx-Gal4 / +; UAS-HFL UAS-Su(H) / + and (C) Bx-Gal4 / +; UAS-HFL UAS-myc-CTD / +. 

The combined over-expression of Hairless with myc-CTD did not lead to a super-repressor (Figure 3C and supporting Figure S1). In contrast, a combination of Hairless with myc-CTD resulted in very mild phenotypes and the flies hatched normally. This result is in line with our model that the CTD overexpression phenotypes are mostly caused by the binding to Hairless since they can be compensated by ectopic Hairless (Figure 3C and supporting Figure S1). Moreover, it supports previous findings that the binding of Su(H) to Hairless is independent of its binding to the DNA [10,48]. The activation of Notch signaling by overexpression of CTD is most likely indirect as the CTD does not contact the DNA [4,5] and hence is probably not involved in the transcriptional activation itself. We thus propose that there are two regulatory modes of Notch signaling regulation: the active mode happens directly on the DNA of Notch target gene promoters whereas the passive mode is based on a competition of the involved molecules, notably of Hairless, distant from the DNA and possibly in the cytoplasm. In fact, genetic data have forestalled this idea a long time ago. The dose sensitivity in particular of *Notch* and *Hairless* mutants is a striking example. Either mutant is haplo-insufficient, resulting in notched wings and bristle loss plus wing vein gaps, respectively [62]. In the combination, the trans-heterozygotes look wild type [62,63], demonstrating the importance of a strictly balanced ratio of the two antagonistic components. 

### In vivo analysis of a *vestigial* reporter in the larval wing disc

**Figure 4:**
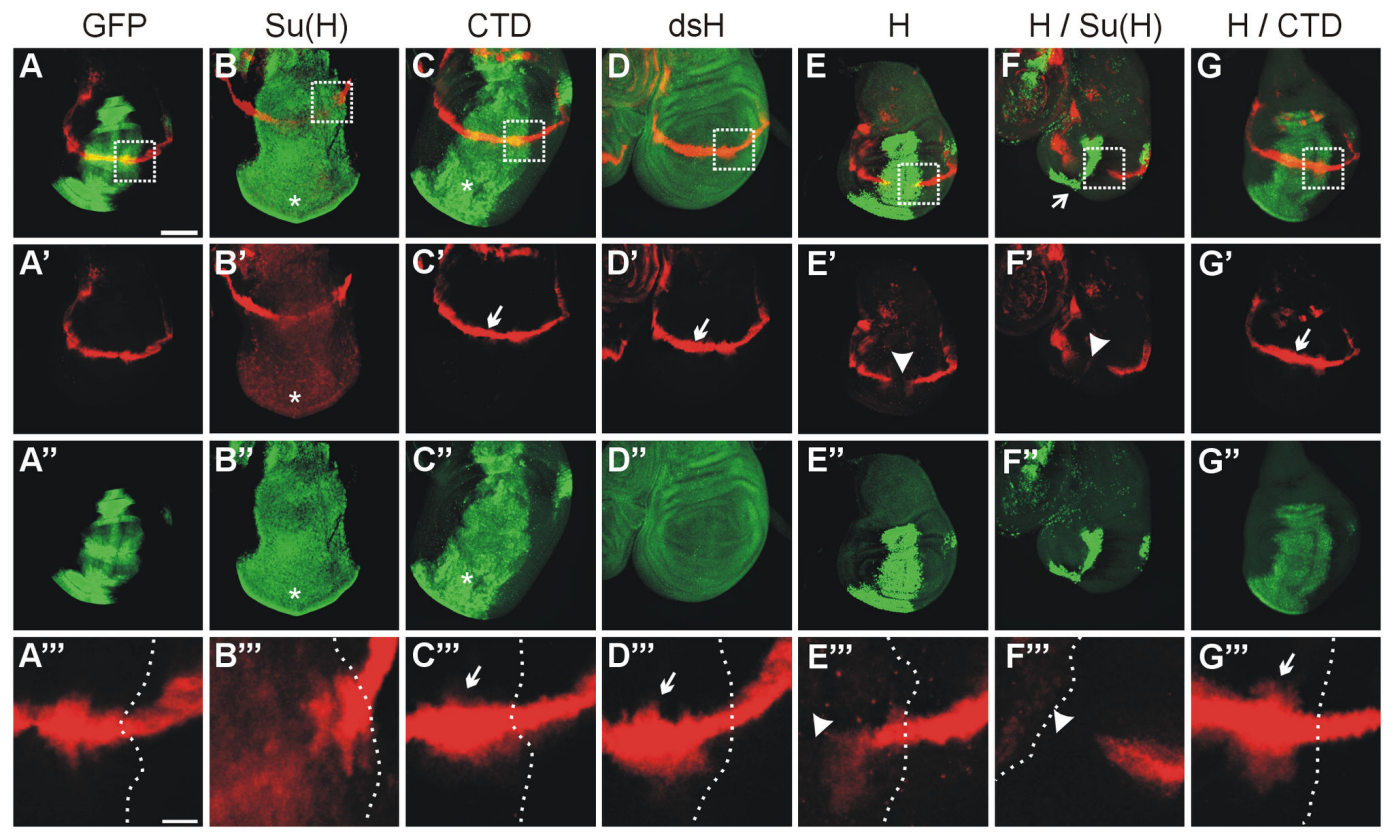
Expression analysis of a vg^BE^ lacZ reporter. Influence on the vestigial boundary enhancer lacZ reporter line (vg^BE^ lacZ) by the ectopic expression of UAS-constructs in the *omb* expression pattern using omb-Gal4. The vg^BE^ lacZ expression is shown in red (A-G’, and A’’’-G’’’), the GFP control is shown in green (A,A’’), as is Hairless (D,D’’, E,E’’), myc-CTD (C,C’’,G,G’’) and Su(H) (B-B’’, F-F’’). A-B’’’ and D-F’’’ were used as reference. A’’’-G’’’ show enlargements boxed in A-G; the dashed line marks the overexpression domain. Compared to control (UAS-GFP) (A-A’’’), overexpression of UAS-Su(H) not only causes overproliferation but also induction of vg^BE^ lacZ expression within the entire overexpression domain (asterisks) (B-B’’’). UAS-myc-CTD overexpression resulted in a mild activation of the vg^BE^ lacZ reporter activity within the omb-expression domain (CTD) (C-C’’’), similar to the downregulation of Hairless by RNA-interference (UAS-dsH) (D-D’’’) (small arrows in C’,C’’’,D’,D’’’). Interestingly, myc-CTD induced tissue overproliferation typical of Notch gain of function (asterisk in C,C’’). Hairless H (UAS-HFL) overexpression repressed the vg^BE^ lacZ reporter (E,E’,E’’’; arrowhead), which was normalized in the presence of UAS-myc-CTD (H / CTD) (G-G’’’). (F-F’’’) The combined ectopic expression of UAS-Su(H) together with Hairless H (UAS-HFL) gives a strong super-repressor phenotype: the vg^BE^ lacZ activity is eliminated in the expression domain (arrowhead in F’, F’’’) and loss of tissue is observed (arrow in F). Discs are oriented with anterior to the left and ventral downwards. Size bar in A (for A-G’’) represents 100 µm; and in A’’’ (for A’’’-G’’’) 20 µm.

In order to gain further insight into the mechanisms underlying CTD activity, we sought to analyze Notch target gene expression. Notch target gene expression was monitored using the vg^BE^ lacZ reporter gene that contains the dorso-ventral boundary element of the *vestigial* gene and only responds to Notch activation [34]. In contrast to other Notch targets like *E*(*spl*) *mbeta* which is repressed by Su(H) overexpression [15], *vestigial* is activated in response to Su(H) allowing to assess activation as well as repression [15,32,34,56,64]. The relevant constructs were overexpressed in the central domain of the wing anlagen, the so-called wing imaginal discs, with the help of the omb-Gal4 driver line to subsequently analyze the expression of the vg^BE^ lacZ reporter. The ectopic expression of myc-CTD with the omb-Gal4 driver line stimulated the wing imaginal discs to over-proliferate just like the ectopic expression of Su(H) (Figure 4A-C’’’). Despite the observed overproliferation, the vg^BE^ lacZ reporter gene expression was only slightly enhanced by ectopic CTD expression (Figure 4C-C’’’). The effect was within the same range resulting from a downregulation of Hairless by RNAi (Figure 4D-D’’’). This is in contrast to the full length Su(H) overexpression, where the vg^BE^ lacZ reporter was induced within the entire omb-expression domain (Figure 4B-B’’’). It was described before that activation of the *vestigial* expression is observed upon relief of Su(H) repression [15,64]. Moreover, Notch target genes, notably the ones controlling cell proliferation, are exquisitely threshold sensitive [65]. Of note the isolated Su(H) and CTD proteins both bind to Hairless NTCT with nearly identical affinity, which is in the nanomolar range [13]. Assuming an accordant binding behavior of the two proteins in vivo, we would expect a similar upregulation of the vg^BE^ lacZ reporter in response to either overexpression. However, this is not the case as the overexpression of Su(H) results in a more potent activation of the vg^BE^ lacZ reporter than that of CTD. One interpretation may be that Su(H) sequesters additional yet unknown Notch repressors that act in the regulation of *vestigial*. We favor the hypothesis that ectopically expressed Su(H) increases the pool of Su(H) molecules available for ICN for activator complex assembly, thereby increasing transcriptional output.

As shown before the co-overexpression of Hairless with Su(H) leads to a very small wing imaginal disc with a complete loss of the vg^BE^ lacZ expression even outside of the omb expression domain [13]. This phenotype is clearly stronger than that after ectopic Hairless expression, where the vg^BE^ lacZ expression is extinguished only in the omb expression domain (Figure 4E-F’’’) [13,32]. In contrast, the combined expression of myc-CTD and Hairless had almost no influence on imaginal disc size and rather enhanced than repressed vg^BE^ lacZ expression (Figure 4G-G’’’). Again this demonstrates the balancing effect of Hairless and myc-CTD supporting the idea that CTD is able to bind and neutralize the repressor Hairless.

### Cell culture activation assay

**Figure 5:**
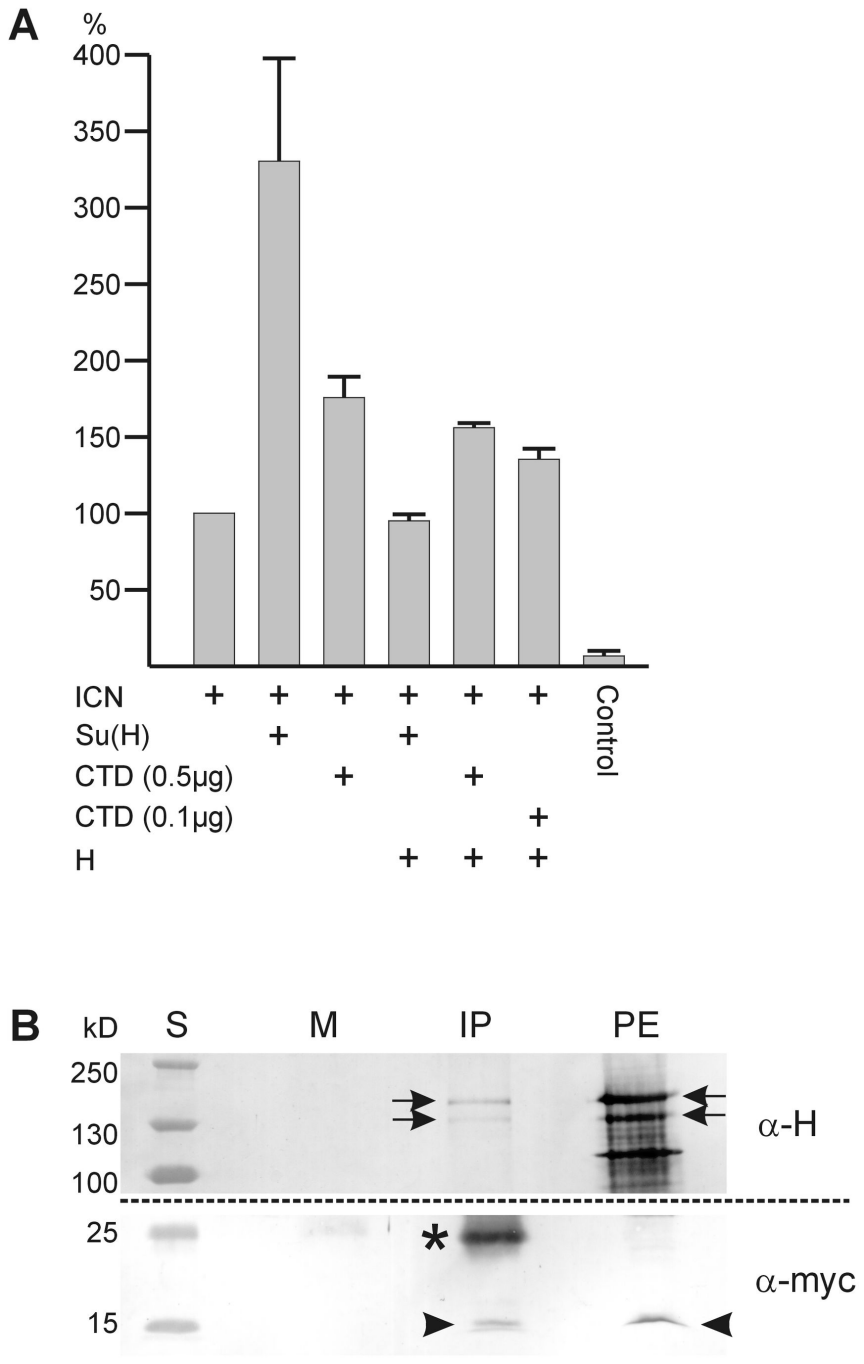
Quantification of the CTD effect in S2 cell culture. A) Transfection of S2 cells with a luciferase reporter gene containing several Su(H) binding sites was performed and activity measured in the presence of the given constructs. The activation by ICN was taken as 100%; this is enhanced about 3.5 fold by addition of Su(H). Addition of myc-CTD activates as well, although to a lesser degree, despite its lack of DNA-binding. The activation by exogenous Su(H) is effectively repressed by exogenous full length Hairless (H). Interestingly, Hairless is less able to suppress activation by myc-CTD. Control is empty pMT-vector. Three independent experiments were sampled, standard deviation is indicated. B) Co-immunoprecipitation was performed on protein extracts from fly heads overexpressing myc-CTD and Hairless in the developing eye (genotype: gmr-Gal4 / +; UAS-HFL UAS-myc-CTD/ +). Anti-myc antibodies were used for precipitation (IP), detection was with anti-Hairless A (α-H; upper blot 7.5% PAGE) and anti-myc antisera (α-myc; lower blot 12% PAGE), respectively. PE, protein extract as input control; M, mock control; S, protein ladder (sizes in kilodaltons, kD). Myc-CTD has an expected size of 13.8 kDa (arrowheads), the two Hairless isoforms are of approximately 150 and 120 kDa (arrows). The smaller bands in the protein extract detected by anti-H antibodies are most likely degradation products; * light chain immunoglobulins.

If CTD regulates Notch activity in a passive manner, we should be able to quantify the effect in cell culture. To this end we used a luciferase reporter gene containing several Su(H) binding sites and assayed its expression in the presence of ICN as shown in Figure 5A [13,29,32]. The Schneider S2 cells are mutant for *Delta* and *Notch*, but neither for *Su*(*H*) nor *Hairless* [13,32,48,66]. ICN stimulates luciferase activity with the help of endogenous Su(H); this activation was taken as 100% (Figure 5A). Additional stimulation to about 350% was measured by adding exogenous full length Su(H) (Figure 5A) and is within the range described earlier [13]. As observed before, this activation is repressed to about the basal level (~94%) by adding exogenous Hairless (Figure 5A) [13]. The extreme repression resembles the effects of the combined overexpression of Su(H) and Hairless during fly development and may be explained by the formation of a super-repressor [11,13,15]. Addition of myc-CTD to Notch enhanced luciferase activity to ~167% (Figure 5A). Transcriptional activation of the luciferase reporter gene by CTD is very unlikely, since the CTD does not contact DNA [4-7]. Probably CTD binds endogenous Hairless, thereby limiting Hairless in the competition with Notch for Su(H). An increase in Notch activity is the consequence. After adding Hairless to the combination of Notch and myc-CTD (0.5 µg) the activation dropped to ~158% but not to the basal level (Figure 5A). We may explain this effect by the different molar ratios, since the Hairless construct (~8kb) is about twice as large as the myc-CTD construct (~4.2 kb). Considering in addition transcription and translation efficiency, myc-CTD may largely outnumber Hairless molecules. Accordingly, a reduction of the myc-CTD concentration (0.1 µg) resulted in a higher repression activity of Hairless. These results indicate that the CTD is able to interfere with Hairless repression, presumably by its direct binding. The in vivo binding of the two proteins was indeed confirmed in a co-immunoprecipitation experiment on fly eyes overexpressing myc-CTD and HFL (Figure 5B). Likewise, it was shown before that Hairless is able to repress Notch activation to about 50% independent of its co-repressors Groucho and CtBP, suggesting competition for Su(H) by the full length Hairless protein [32].

### Nuclear accumulation of Su(H) and myc-CTD proteins responds to Hairless

**Figure 6:**
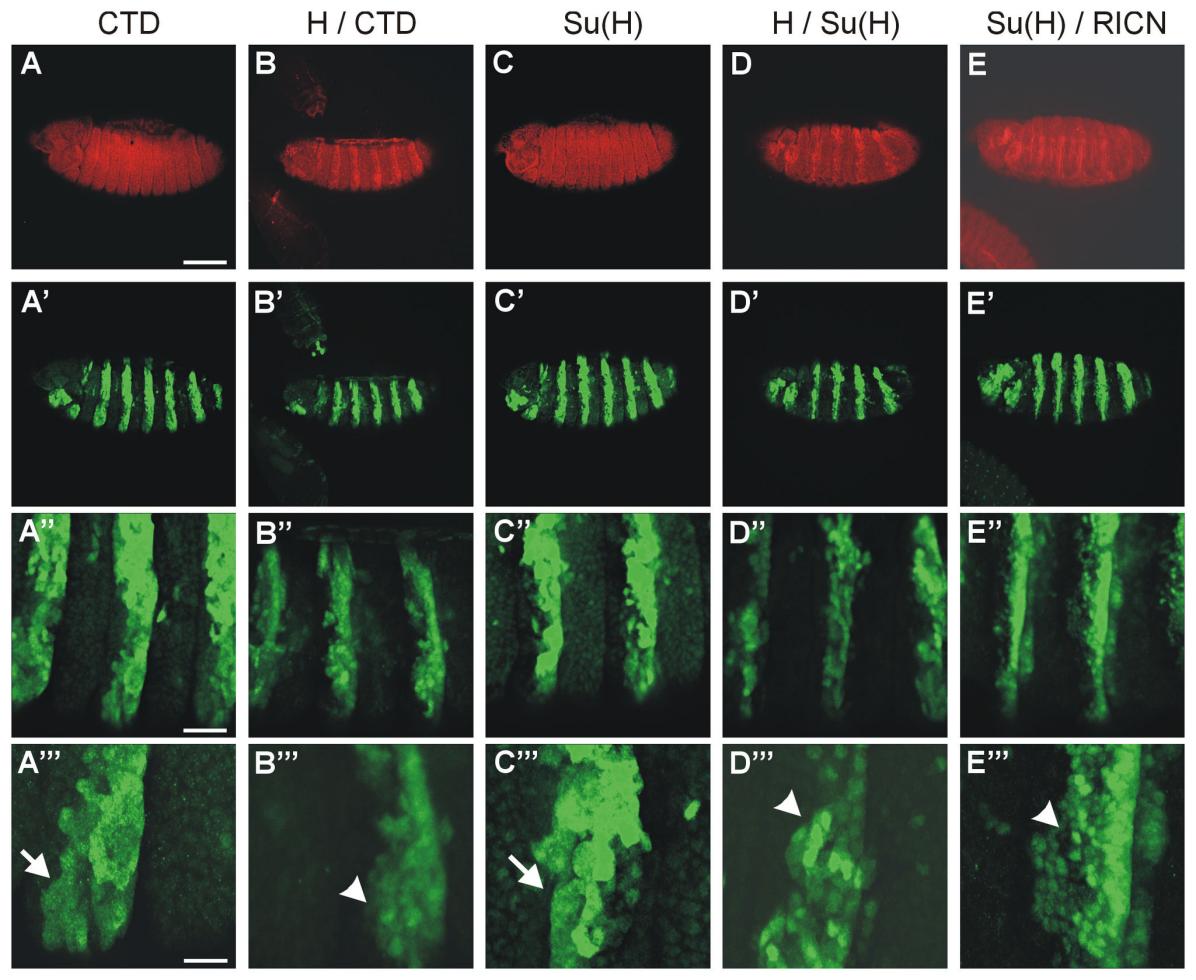
Nuclear localization of CTD and Su(H). Either CTD or Su(H) were expressed in the presence or absence of either Hairless or RICN in a zebra pattern in the embryo using prd-Gal4 as indicated. The upper panel (A-D) shows Hairless protein in red except for (E), where the Notch intracellular domain was detected. The lower panels show expression of Su(H) and myc-CTD (green) as indicated. (A’’’-E’’’) are enlargements of (A’-E’’). (A-A’’’) Whereas myc-CTD is mostly found in the cytoplasm (CTD, arrow in A’’’), it is predominantly nuclear when coexpressed with Hairless (H / CTD; arrowhead in B’’’). Su(H) is likewise nuclear and cytoplasmic upon overexpression (arrow in C’’’), however appears enriched in the nucleus (arrowhead in D’’’ and E’’’) when co-expressed with either Hairless (H / Su(H)) or RICN (Su(H) / RICN). Genotypes are: prd-Gal4 / UAS-myc-CTD / + (A-A’’’), prd-Gal4 / UAS-HFL UAS-myc-CTD / + (B-B’’’); prd-Gal4 / UAS-Su(H) / + (C-C’’’), prd-Gal4 / UAS-HFL UAS-Su(H) / + (D-D’’’), prd-Gal4 / +; UAS-Su(H) UAS-RICN/ + (E-E’’’). Size bars in A-E, A’-E’ 100 µm; in A’’-E’’ 25 µm; in A’’’-E’’’ 10 µm.

In an attempt to analyze the subcellular distribution and availability, ectopic expression of the given constructs was enforced in a striped pattern in embryos using the prd-Gal4 driver line (Figure 6). Both, myc-CTD and Su(H) overexpressed proteins showed a uniform abundance in the expressing cells (both in green, see Figure 6), which is in accordance with published data [18]. Endogenous Hairless protein is both cytoplasmic and nuclear, but strongly accumulated in the nucleus in response to overexpression (in red in Figure 6) as described before [19]. Nuclear enrichment of either Su(H) or CTD was likewise observed, when they were co-expressed together with Hairless (Figure 6B-B’’’ and D-D’’’). Based on the published data we conclude that the Hairless protein is involved in shuttling Su(H) and CTD into the nucleus, suggesting that the interaction between Hairless and Su(H) can take place in the cytoplasm [18,19]. By influencing the levels of available Su(H), Hairless may at least in part act as an indirect Notch antagonist. There are several possibilities as to the underlying mechanisms including the stability of Su(H) or its subcellular distribution that may be affected by Hairless.

In contrast to Su(H), Hairless contains several potential nuclear localization signals [39,47,48]. In the absence of Hairless, as is the case in *Hairless* mutant cell clones, Su(H) is less abundant in the nucleus as in the wild type situation [19]. However, a nuclear enrichment of Su(H) is observed upon ectopic expression of Hairless [19]. The same holds true for CTD indicating that Hairless can contact CTD in the cytoplasm and cause its nuclear import. A likewise nuclear accumulation of Su(H) is detected in response to the co-overexpression of the intracellular domain of Notch (Figure 6E-E’’’), in accordance with a role for ICN in the shuttle process that has been shown before [15,17,18]. Whereas Notch protein is predominantly at the plasma membrane [67,68], its nuclear shuttling was visualized by life imaging using tagged constructs [69]. Further work will be required to resolve the respective contribution of the co-activator ICN and the co-repressor Hairless for Su(H) nuclear import and availability on the DNA.

## Supporting Information

Figure S1 **CTD overexpression causes Notch gain of function phenotypes also during eye development.** Overexpression of UAS-constructs as indicated was induced in the differentiation eye field using the Gmr-Gal4 driver line. The colored pictures are taken with the ES120 camera, the grey pictures from a scanning electron microscope. Eye overgrowth and glossy appearance is typical of Notch gain of function [56-58], and is observed upon overexpression of myc-CTD or Su(H). Notch loss of function, as seen upon overexpression of Hairless (H), is typified by small rough eyes [59-61]. Note complete loss of eyes when the super-repressor is formed upon combined expression of Su(H) and Hairless (H / Su(H)). These animals die as pharate adults. Wild type or near wild type phenotypes are seen in the GFP control and upon combined overexpression of myc-CTD and Hairless (H / CTD).
(TIF)

Figure S2 **Overexpression of Su(H)^E446K^ causes mild Notch gain of function phenotypes during bristle development.** Compared with a control fly (A), overexpression of Su(H)^E446K^ (B) causes a partial shaft to socket transformation, resulting in a double socket phenotype. Genotypes are in (A) Bx-Gal4 / +; UAS-GFP / + and in (B) Bx-Gal4 / +; UAS-Su(H)^E446K^ / +.
(TIF)
