# Supplementary figures and images for: Gain of Function Notch Phenotypes Associated with Ectopic Expression of the Su(H) C-Terminal Domain Illustrate Separability of Notch and Hairless-Mediated Activities
Source: PLoS One. 2013 Nov 25;8(11):e81578. doi: 10.1371/journal.pone.0081578 (PMC3839874; doi:10.1371/journal.pone.0081578)

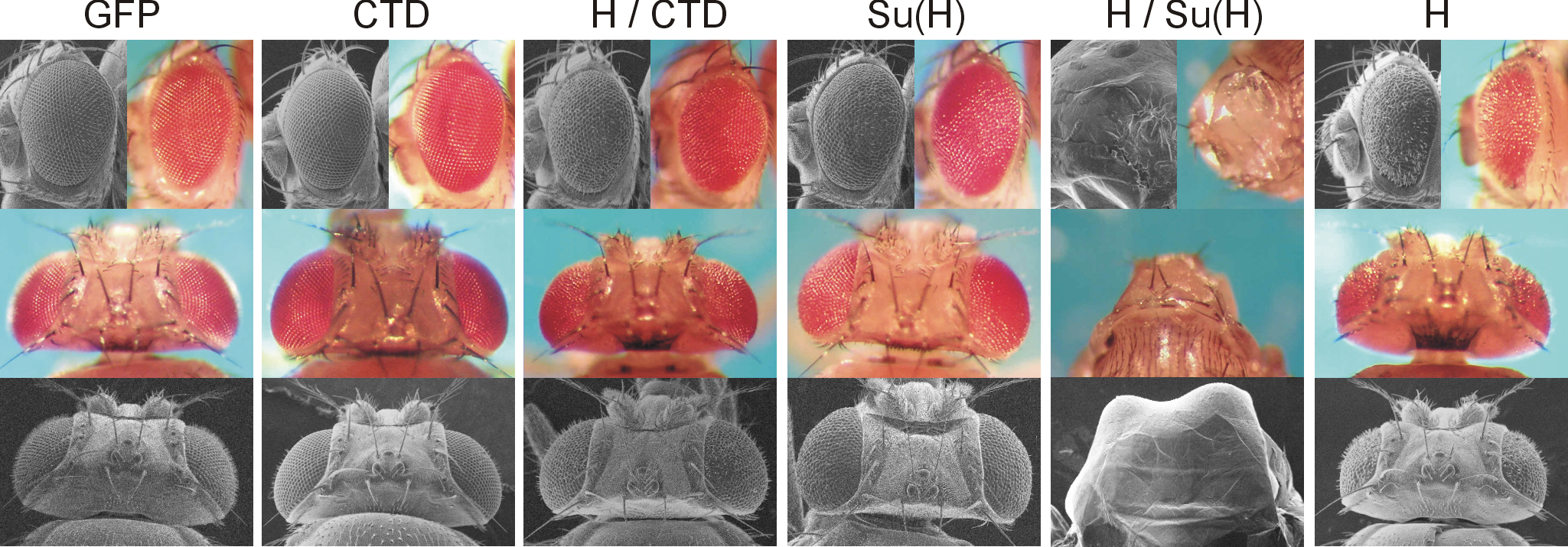

Supplement: Figure S1 — CTD overexpression causes Notch gain of function phenotypes also during eye development. Overexpression of UAS-constructs as indicated was induced in the differentiation eye field using the Gmr-Gal4 driver line. The colored pictures are taken with the ES120 camera, the grey pictures from a scanning electron microscope. Eye overgrowth and glossy appearance is typical of Notch gain of function [56-58], and is observed upon overexpression of myc-CTD or Su(H). Notch loss of function, as seen upon overexpression of Hairless (H), is typified by small rough eyes [59-61]. Note complete loss of eyes when the super-repressor is formed upon combined expression of Su(H) and Hairless (H / Su(H)). These animals die as pharate adults. Wild type or near wild type phenotypes are seen in the GFP control and upon combined overexpression of myc-CTD and Hairless (H / CTD). (TIF) [file pone.0081578.s001.tif]

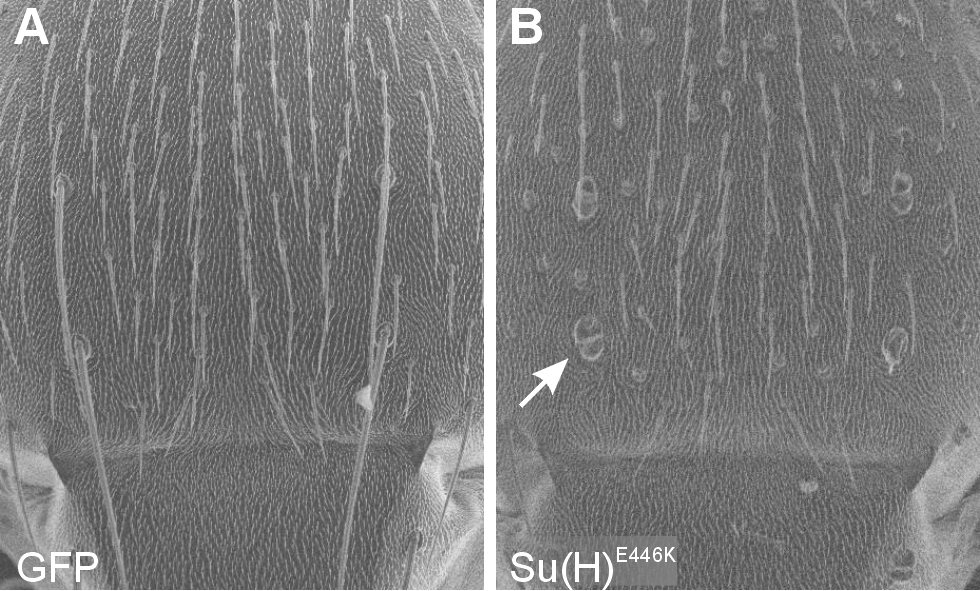

Supplement: Figure S2 — Overexpression of Su(H)E446K causes mild Notch gain of function phenotypes during bristle development. Compared with a control fly (A), overexpression of Su(H)E446K (B) causes a partial shaft to socket transformation, resulting in a double socket phenotype. Genotypes are in (A) Bx-Gal4 / +; UAS-GFP / + and in (B) Bx-Gal4 / +; UAS-Su(H)E446K / +. (TIF) [file pone.0081578.s002.tif]
